# Supplementary material for: AI-enabled virtual spatial proteomics from histopathology for interpretable biomarker discovery in lung cancer
Source: Nat Med. 2026 Jan 5;32(1):231–44. doi: 10.1038/s41591-025-04060-4 (PMC12823406; doi:10.1038/s41591-025-04060-4)
Supplement: Supplementary file 1 — Supplementary Figs. 1–30, Tables 2–4, results, methods and references. [file 41591_2025_4060_MOESM1_ESM.pdf]

nature medicine

Supplementary information https://doi.org/10.1038/s41591-025-04060-4

AI-enabled virtual spatial proteomics from histopathology for interpretable biomarker discovery in lung cancer

In the format provided by the authors and unedited

1. Supplementary Results

Subcellular stratification. To examine whether prediction accuracy varies by subcellular localization, we divided the 40 protein markers into nuclear, cytoplasmic, and membrane groups, and calculated the average Pearson correlation for each category. As shown in Extended Data Fig. 4, HEX performed similarly across all three compartments, with slightly higher accuracy for nuclear markers. These results indicate that HEX generalizes well across proteins with diverse localization patterns.

Prognostic robustness in NSCLC. We extended our analyses to include patients across all disease stages. Our analyses again demonstrated that MICA-derived risk scores remained a strong and independent predictor of recurrence and survival across validation cohorts (Supplementary Fig. 7). Furthermore, subgroup analyses revealed that the MICA model retained robust prognostic performance when stratifying patients by age, sex, and tumor grade within each cohort (Supplementary Fig. 8-12).

We further evaluated the robustness of MICA through cross validation separately within each of three largest cohorts: NLST, TCGA, and PLCO. The results are consistent with our previous findings in independent validation with slightly better prognostic performance (Supplementary Fig. 13).

We compared our early-fusion MICA approach with a late-fusion deep learning method, i.e., PORPOISE[1]. In both independent validation and cross-validation settings, MICA consistently outperformed PORPOISE for predicting prognosis as measured by c-index. The performance gains achieved by MICA were statistically significant across all comparisons ($P < 0.05$, Supplementary Fig. 14), highlighting the advantage of the early fusion strategy with co-attention for multi-modal data integration.

Pan-cancer prognostic generalization. To assess the generalizability and clinical utility of HEX-derived virtual spatial proteomics beyond lung cancer, we performed prognostic modeling across 12 additional cancer types, including breast, colorectal, liver, pancreas, bladder, kidney cancers in TCGA (n = 5,019 patients). For each cancer type, we performed five-fold cross validation and compared three different models: (i) an H&E-only baseline using MUSK-derived features, (ii) a virtual CODEX-only model using HEX-inferred protein maps, and (iii) the full MICA model integrating both modalities.

As shown in Supplementary Fig. 16, the full MICA model consistently outperformed both unimodal baselines models across all 12 cancer types. On average, MICA achieved a c-index of 0.73, compared to 0.67 for the H&E-only model and 0.66 for the virtual CODEX-only model (overall P < 0.001). Kaplan-Meier analysis showed statistically significant risk

stratification with MICA across all 12 cancer types (hazard ratio range: 1.58-13.3; log-rank $P \leq 0.0001$; Supplementary Fig. 15). Together, these findings demonstrate that multimodal integration using HEX-derived virtual spatial proteomics provides significant improvements over H&E-based models for prognosis prediction across diverse tumor types.

Clinical utility and reclassification. We assessed how HEX–MICA would improve patient stratification and influence treatment decisions. Compared to standard biomarker PD-L1, the HEX model correctly reclassified 4 out 35 (11%) patients as true responders; at the same time, HEX also correctly reclassified 6 out 55 (11%) patients as true non-responders, resulting in a positive net reclassification index of 22%. At a specificity level of 90%, HEX achieved a sensitivity of 61% for identifying responders, much higher than PD-L1 (19%) and TMB (10%) (Supplementary Fig. 21). Notably, HEX identified clinically relevant subgroups: among patients with PD-L1 ≥50%, HEX separated responders from non-responders with median PFS of 20 vs. 4 months, who may benefit from combination ICI and chemotherapy (Fig. 6c). For patients with intermediate PD-L1 expression (≥1% and <50%), HEX also significantly stratified patients for PFS (HR = 3.57, P = 0.0009). Among PD-L1-negative patients, HEX distinguished responders from non-responders (median PFS: 13 vs. 4 months), highlighting the need for novel combination therapies. Lastly, HEX identified a subgroup (29%) of EGFR-mutant patients who may benefit from ICIs despite these tumors being refractory as a whole (median PFS: 8 vs. 4 months, Supplementary Fig. 22).

Comparison with spatial transcriptomics-based prediction. To assess spatial proteomics relative to transcriptomics-based approaches, we compared HEX against two state-of-the-art spatial transcriptomics (ST) prediction models—BLEEP[2] and OmiCLIP[3]—using the Visium lung cancer ST dataset[4]. The ST models were trained to predict the 2,000 highly variable genes, including 18 that overlapped with protein markers used in our analysis. HEX achieved substantially higher Pearson and Spearman correlations across the full marker panel and the overlapping subset (Supplementary Fig. 26). These findings demonstrate that protein prediction from H&E yields a much higher accuracy than transcript-level prediction, likely due to the improved stability of protein targets in clinical samples and their more direct relations to morphological phenotypes and biological functions.

We next assessed clinical utility of both approaches by applying the same MICA to two input combinations: H&E plus virtual ST versus H&E plus virtual CODEX. In the Stanford-IO cohort, MICA-CODEX significantly outperformed MICA-ST (2,000 genes) for predicting objective response (AUC = 0.82 vs. 0.75, P < 0.001) as well as PFS (C-index = 0.72 vs. 0.65, P < 0.001; Supplementary Fig. 27). Taken together, these results demonstrate that

HEX not only yields more accurate spatial proteomic maps than transcriptomics-based approaches, but also provides greater clinical utility in predicting treatment outcomes.

Scaling laws. We systematically evaluated the influence of training dataset size on HEX prediction performance using incrementally larger subsets of the Stanford-WSI dataset (2, 4, 8, and 10 WSIs). Since we used matched histopathology and spatial proteomics data, each WSI comprises a large number of patches (75,500 on average) with individually labeled protein expressions, resulting in a substantial training dataset at the patch level. Our analysis demonstrated that the accuracy of protein prediction increases with more WSIs used for training. We observed significant improvements when expanding from 2 to 4 WSIs (Δ Pearson r = +0.211, Supplementary Fig. 28), with diminishing gains from 8 to 10 WSIs (Δ Pearson r = +0.013). This indicates that a scaling law does existing within our dataset, where the protein prediction accuracy increases with larger training datasets. With a sufficiently large training set, the improvement in model performance seems to be incremental beyond 500,000 image patches.

We also assessed how the number of protein markers impacts downstream clinical predictions. To do this, we constructed reduced marker panels of size K = 5, 10, and 20, where the markers were selected based on their association with outcome in the NLST training cohort. We then re-trained the MICA prognostic model on NLST and evaluated performance on four independent validation cohorts (TCGA-LUNG, PLCO-LUNG, Stanford-TMA, and TA-TMA), as well as via cross validation on the Stanford immunotherapy cohort. Across these cohorts, we observed a clear scaling law where the prognostic accuracy increases with larger panel sizes from 5 to 40 markers (Supplementary Fig. 29). Compared with H&E alone, models using the first five protein markers improved the C-index by 7%, the next five by an additional 4% across validation cohorts. Beyond 20 markers, the models showed incremental but diminishing gains in performance.

2. Supplementary Methods

Comparison with spatial transcriptomics prediction models. We compared HEX with two state-of-the-art H&E-to-spatial transcriptomics (ST) prediction models: BLEEP and OmiCLIP. BLEEP leverages contrastive learning to align histology images with spatial gene expression profiles[2], while OmiCLIP is a visual-omic foundation model pretrained on a large corpus of paired H&E–ST data[3]. For evaluation, we used a Visium lung cancer dataset[4], comprising 20 paired H&E and spatial transcriptomics slides. Of these, 17 slides were used for training and 3 were held out for evaluation. BLEEP was trained using its official implementation, and OmiCLIP was configured following the HEST-Benchmark framework[5]. Both ST-based models were trained to predict the 2,000 highly variable genes, including 18 genes overlapping with the protein markers used in our study. All predicted gene profiles were used to evaluate both biomarker prediction accuracy and downstream clinical performance.

3. Supplementary References

1. Chen, R.J., *et al.* Pan-cancer integrative histology-genomic analysis via multimodal deep learning. *Cancer cell* **40**, 865-878. e866 (2022).
2. Xie, R., *et al.* Spatially resolved gene expression prediction from histology images via bi-modal contrastive learning. *Advances in Neural Information Processing Systems* **36**, 70626-70637 (2023).
3. Chen, W., *et al.* A visual–omics foundation model to bridge histopathology with spatial transcriptomics. *Nature Methods*, 1-15 (2025).
4. Madissoon, E., *et al.* A spatially resolved atlas of the human lung characterizes a gland-associated immune niche. *Nature genetics* **55**, 66-77 (2023).
5. Jaume, G., *et al.* Hest-1k: A dataset for spatial transcriptomics and histology image analysis. *Advances in Neural Information Processing Systems* **37**, 53798-53833 (2024).

4. Supplementary Figures

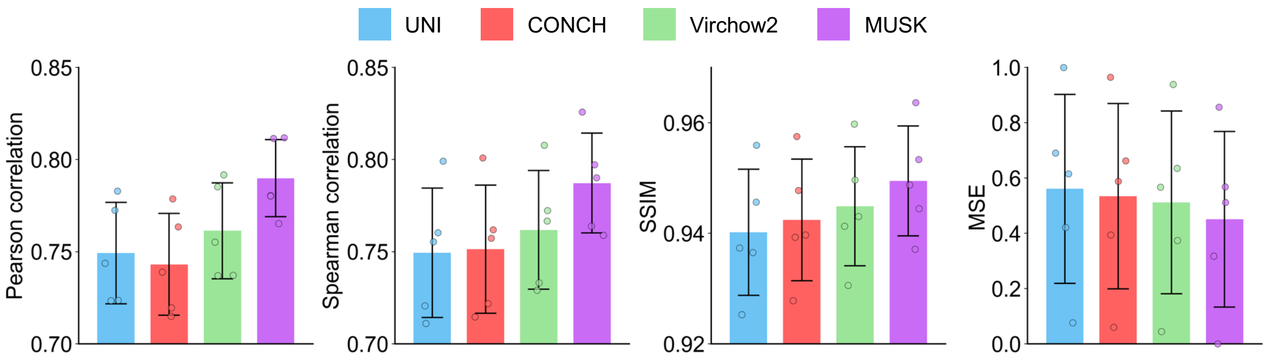

Supplementary Fig. 1 | Performance comparison of foundation model backbones for HEX using five-fold cross-validation on Stanford-WSI cohort. Among the tested backbones, HEX initialized with MUSK achieved the highest predictive accuracy. Bars represent the mean across five-fold cross-validation on the Stanford-WSI dataset (n = 10 WSIs); dots show individual folds and error bars indicate standard deviation.

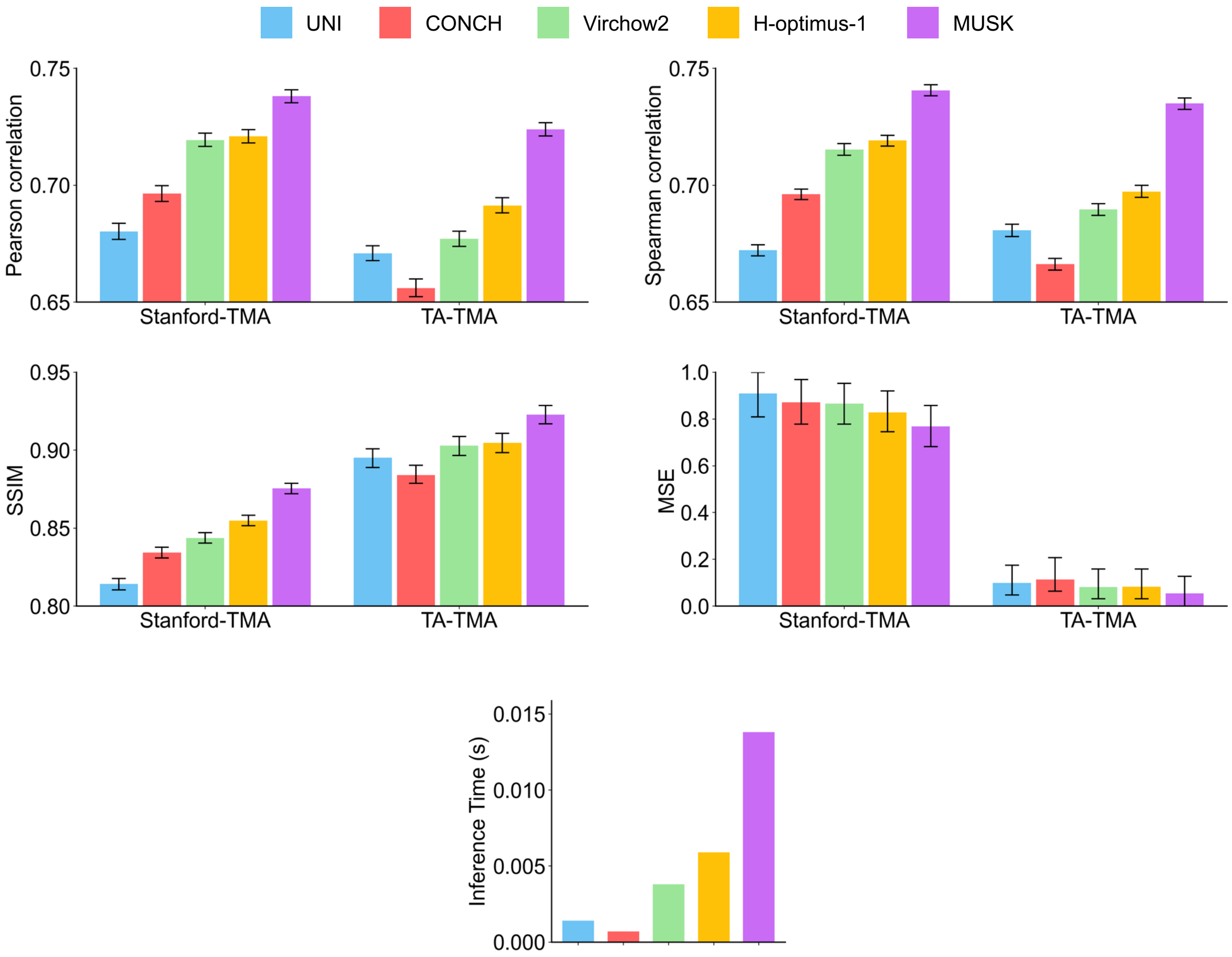

Supplementary Fig. 2 | Performance comparison of foundation model backbones for HEX on independent validation cohorts. MUSK-based HEX consistently outperformed other backbones, achieving the highest predictive accuracy across external datasets (Stanford-TMA, n = 264 cores; TA-TMA, n = 108 cores). Models based on CONCH, UNI, Virchow2, and H-optimus-1 show varying levels of performance and speed, reflecting different trade-offs between accuracy and computational efficiency. Bars represent point estimates and error bars indicate 95% bootstrap CIs (n = 1,000 resamples).

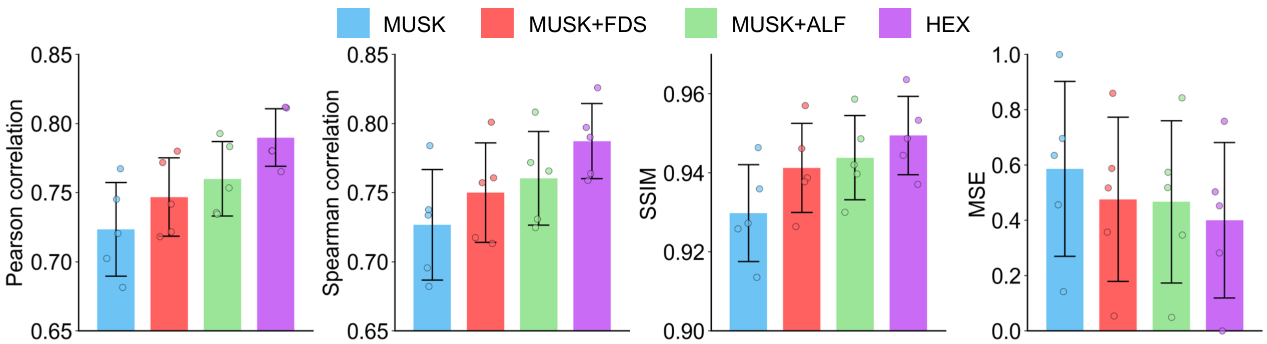

Supplementary Fig. 3 | Performance comparison when using different training strategies for HEX on Stanford-WSI cohort. Ablation experiments were conducted by removing either feature distribution smoothing or adaptive loss function from the full HEX model. In both cases, performance declined notably across all evaluation metrics, highlighting the necessity of these components for achieving robust and generalizable predictions on the training cohort. Bars represent the mean across five-fold cross-validation on the Stanford-WSI dataset (n = 10 WSIs); dots show individual folds and error bars indicate standard deviation.

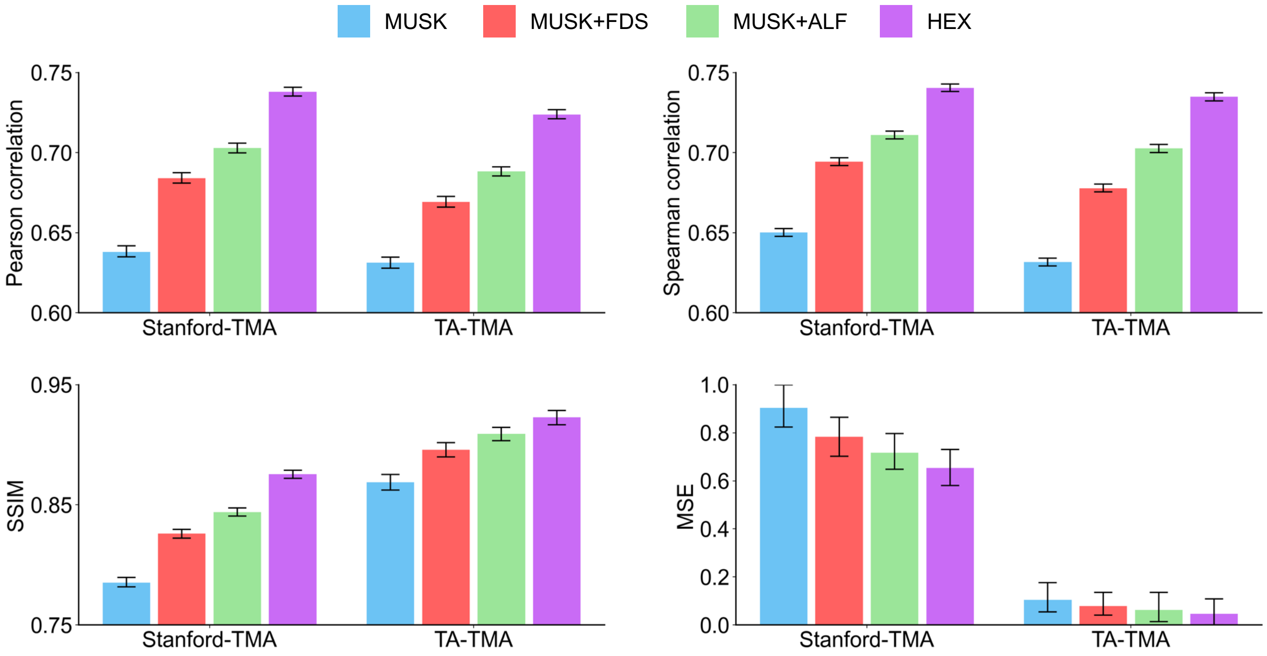

Supplementary Fig. 4 | Performance comparison when using different training strategies for HEX on independent validation cohorts. Removing either feature distribution smoothing or adaptive loss function significantly degraded performance across two independent cohorts (Stanford-TMA, n = 264 cores; TA-TMA, n = 108 cores), reinforcing the critical role of these components in enabling generalization across distinct datasets. Bars represent point estimates and error bars indicate 95% bootstrap CIs (n = 1,000 resamples).

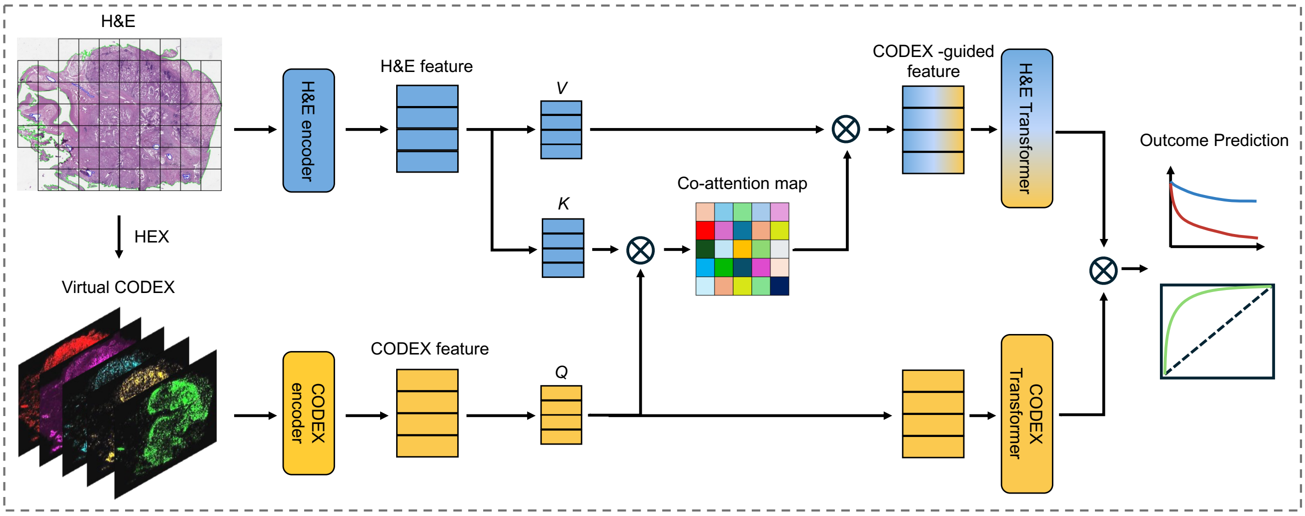

Supplementary Fig. 5 | Overview of the MICA framework. First, a pathology foundation model is used to extract features from H&E images, forming histology feature bags. Second, DINOv2 is applied to CODEX images to generate corresponding CODEX feature bags. Third, CODEX-guided co-attention layers learn cross-modal interactions between histology and CODEX features. Finally, two modality-specific multiple-instance learning Transformers with global average pooling aggregate the features for outcome prediction.

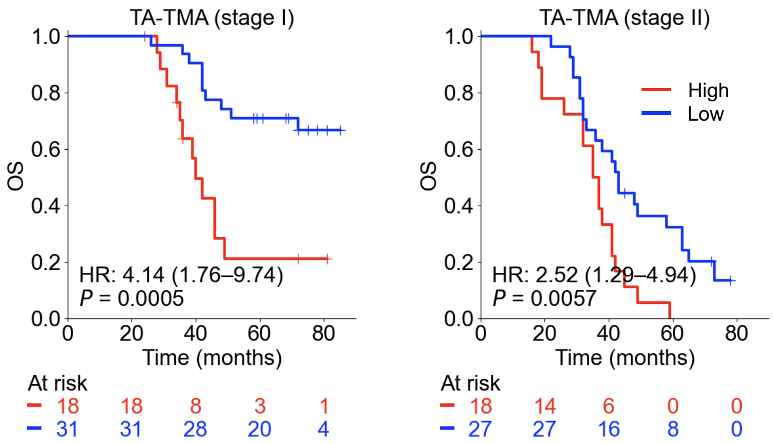

Supplementary Fig. 6 | Kaplan–Meier analysis of MICA-predicted risk groups in stage I–II patients from the TA-TMA cohort. MICA significantly stratified patients by OS. P values were calculated by two-sided log-rank test; risk groups were defined based on the median cutoff derived from the entire TA cohort.

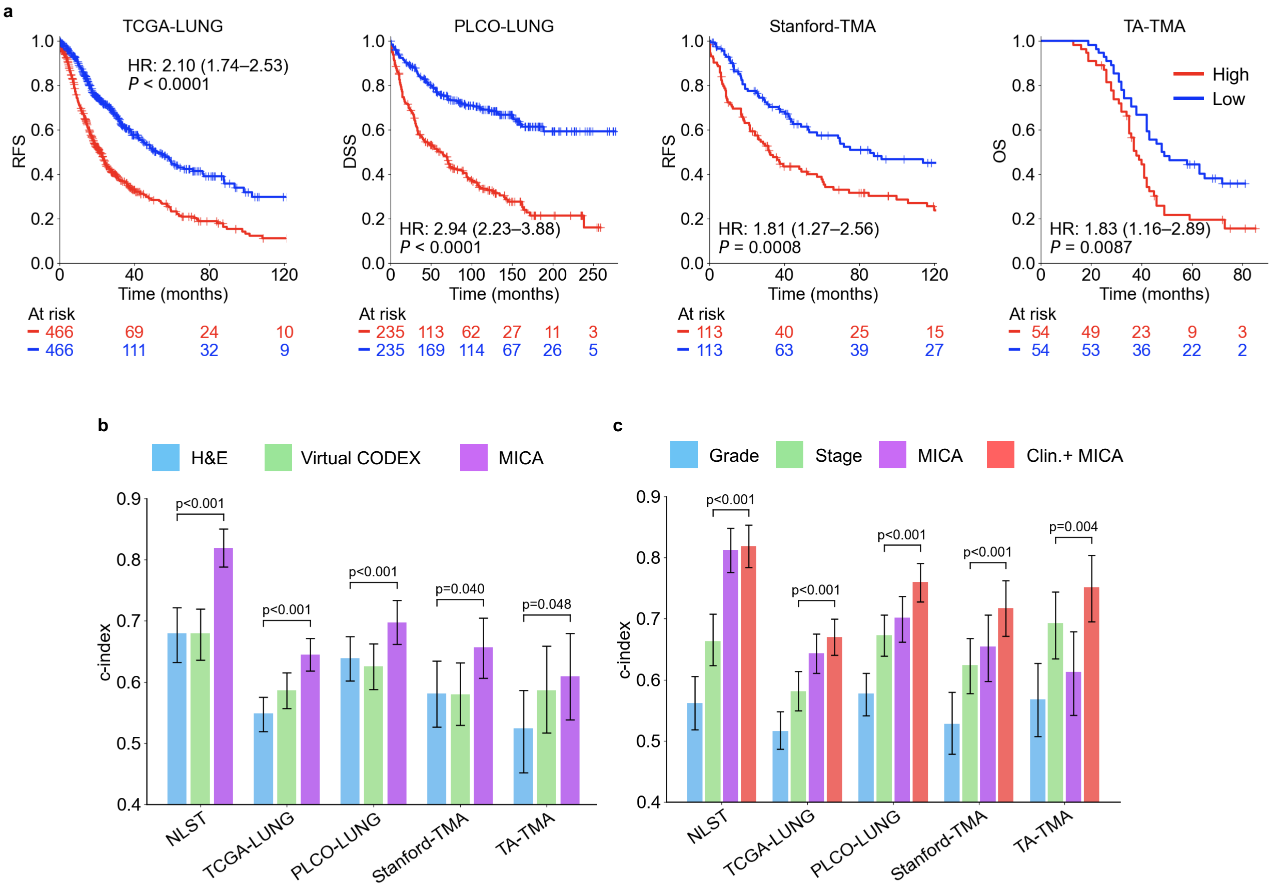

Supplementary Fig. 7 | MICA improves prognosis prediction across all stages of NSCLC. **a**, Kaplan–Meier analysis of MICA-predicted risk groups in patients with NSCLC across all stages. MICA significantly stratified patients into high- and low-risk groups for recurrence and survival across four independent validation cohorts. Risk groups were defined using the median cutoff within each cohort. P values were calculated using a two-sided log-rank test. **b**, Performance comparison of prognostic models across five NSCLC cohorts. **c**, Comparison of MICA-derived risk prediction with traditional clinical features. In **b** and **c**, bars represent point estimates and error bars indicate 95% bootstrap CIs (n = 1,000 resamples). P values were calculated by a two-sided paired bootstrap and were unadjusted.

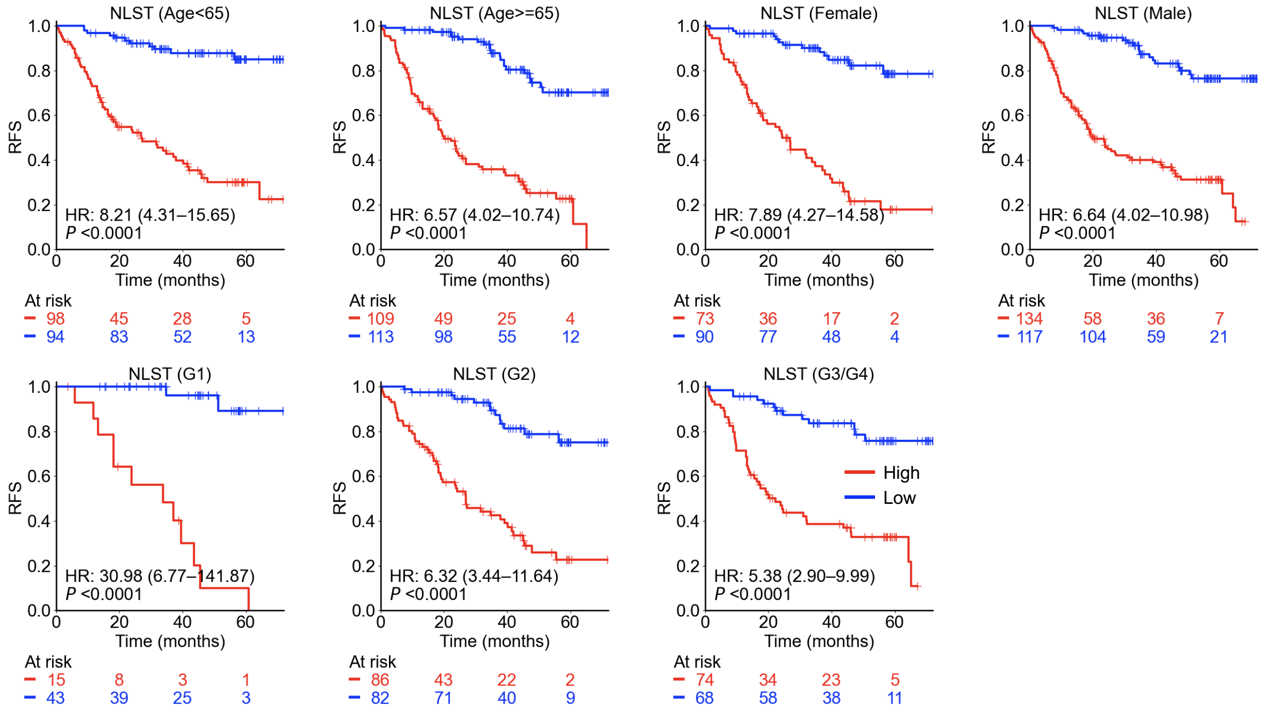

Supplementary Fig. 8 | Kaplan–Meier analysis of MICA risk groups in NLST subgroups. MICA significantly stratified patients by RFS across clinical subgroups. P values were calculated using two-sided log-rank tests; risk groups were defined by the median cutoff.

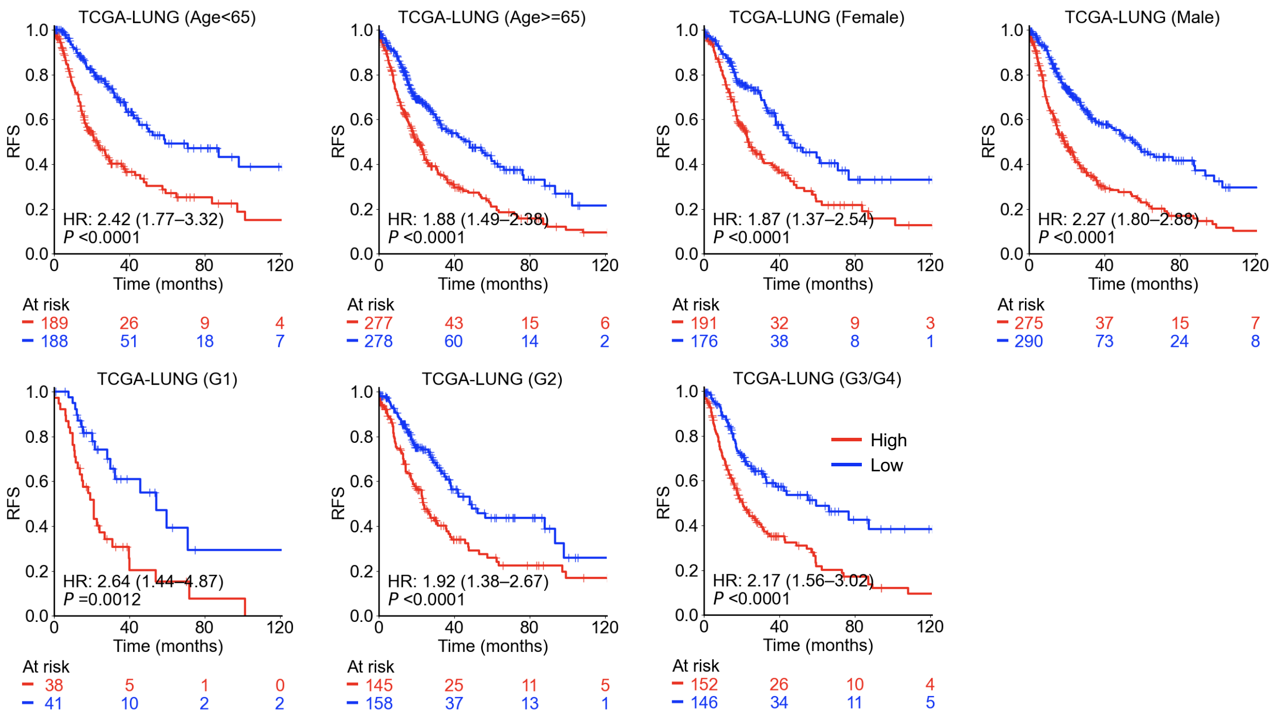

Supplementary Fig. 9 | Kaplan–Meier analysis of MICA risk groups in TCGA subgroups. MICA significantly stratified patients by RFS across clinical subgroups. P values were calculated using two-sided log-rank tests; risk groups were defined by the median cutoff.

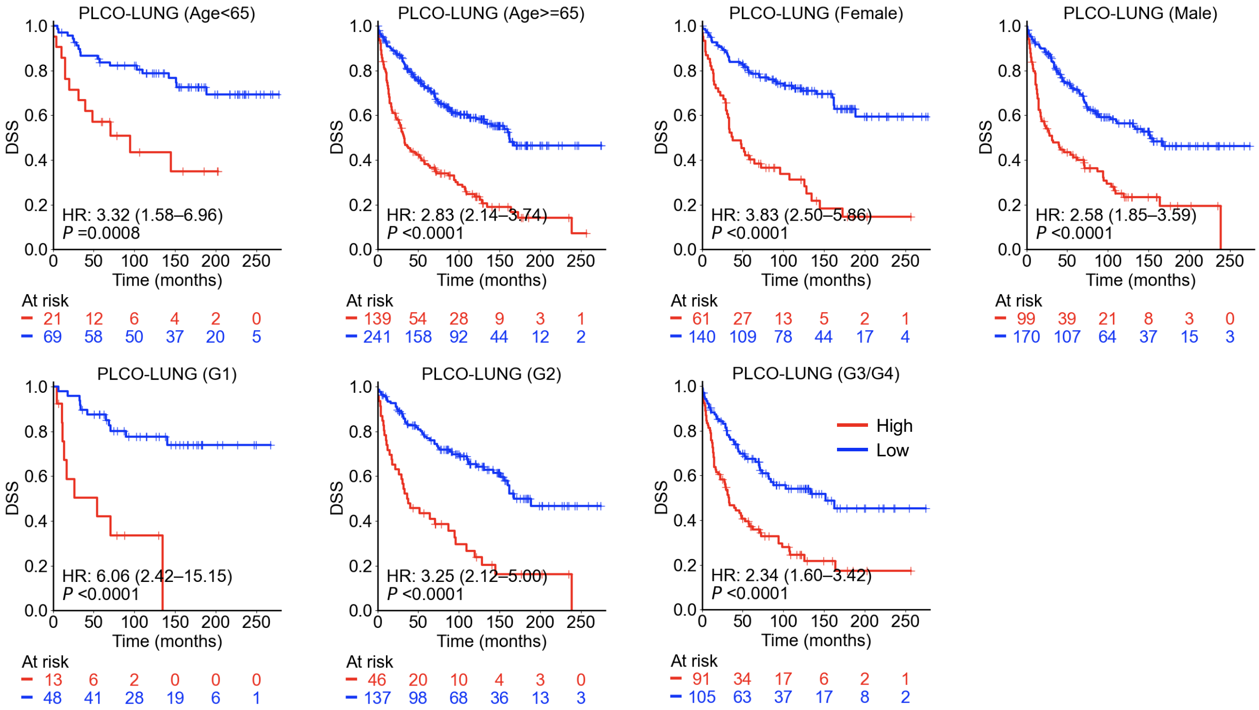

Supplementary Fig. 10 | Kaplan–Meier analysis of MICA risk groups in PLCO subgroups. MICA significantly stratified patients by DSS across clinical subgroups. P values were calculated using two-sided log-rank tests; risk groups were defined by the upper tertile cutoff.

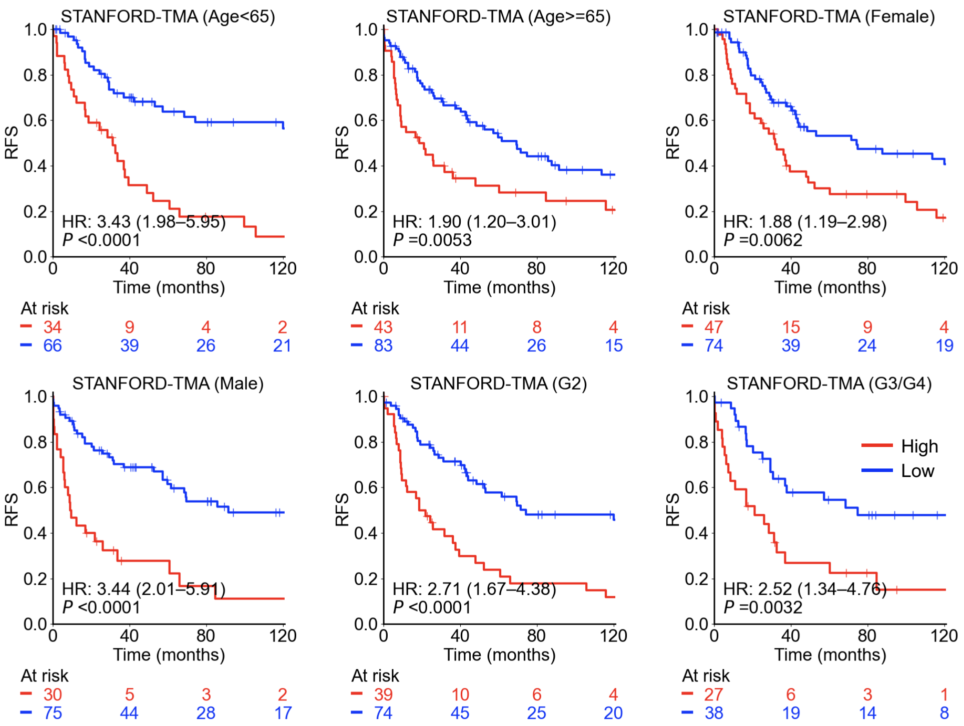

Supplementary Fig. 11 | Kaplan–Meier analysis of MICA risk groups in Stanford-TMA subgroups. MICA significantly stratified patients by RFS across clinical subgroups. P values were calculated using two-sided log-rank tests; risk groups were defined by the upper tertile cutoff.

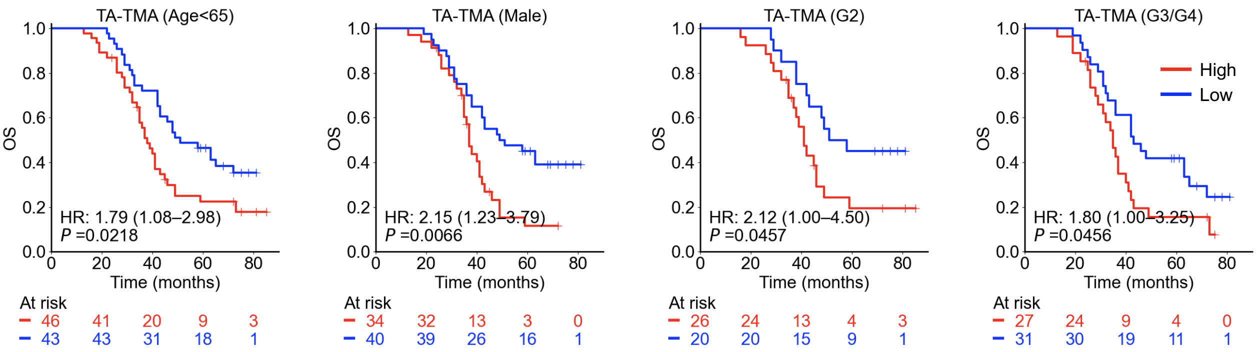

Supplementary Fig. 12 | Kaplan–Meier analysis of MICA risk groups in TA-TMA subgroups. MICA significantly stratified patients by RFS across clinical subgroups. P values were calculated using two-sided log-rank tests; risk groups were defined by the median cutoff.

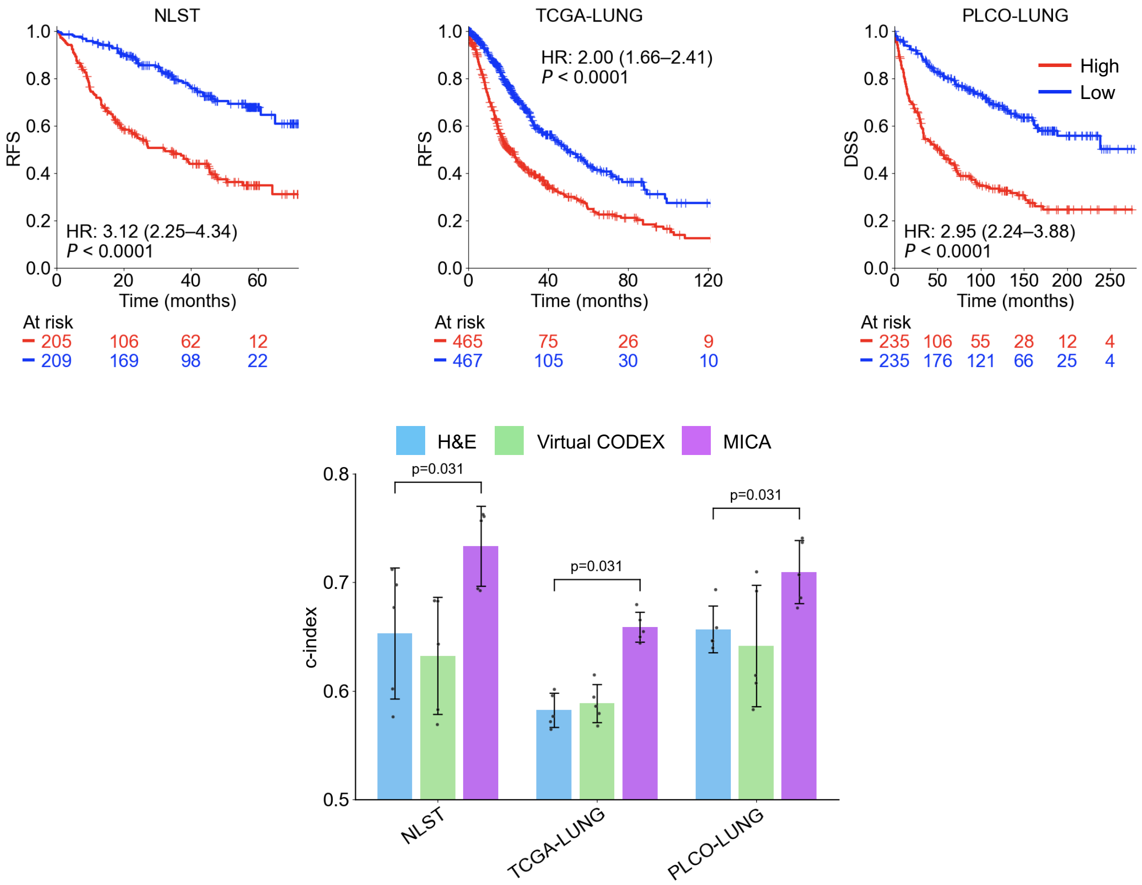

Supplementary Fig. 13 | Cross-validation performance of MICA within the three largest NSCLC cohorts. **a**, Kaplan–Meier analysis of MICA-predicted risk groups within NLST, TCGA, and PLCO cohorts. Patients were significantly stratified by RFS or DSS using cohort-specific median cutoffs. P values were calculated using two-sided log-rank tests. **b**, Bar plots showing mean c-index from five-fold cross-validation within each cohort, with error bars indicating standard deviation. P values were calculated using two-sided Wilcoxon signed-rank tests.

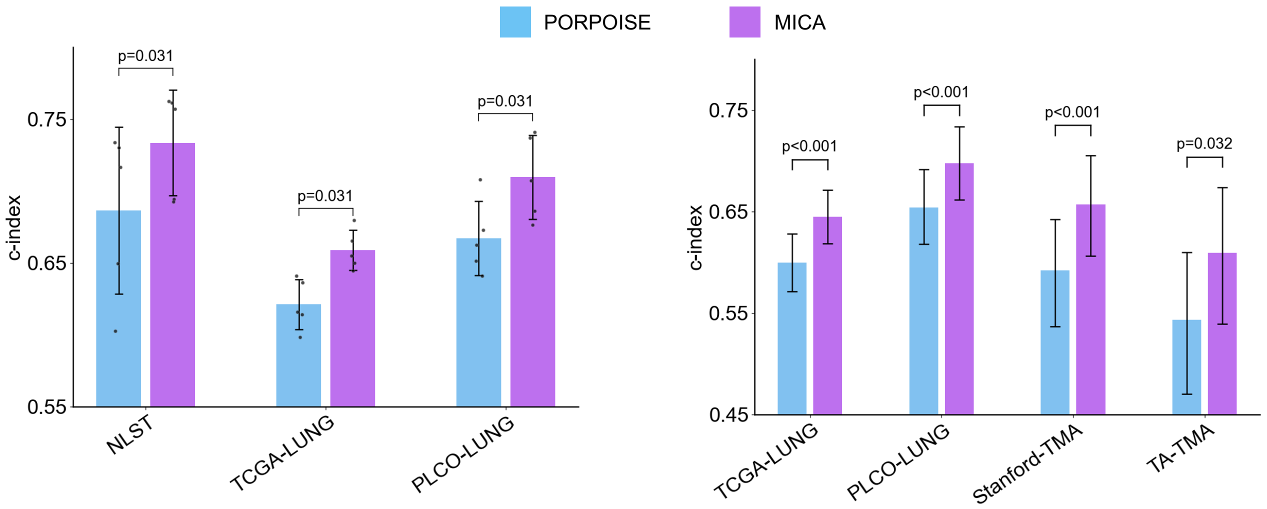

Supplementary Fig. 14 | Performance comparison between MICA and PORPOISE.
a, Cross-validation performance across three NSCLC cohorts. Bar plots show the mean c-index from five-fold cross-validation, with error bars indicating standard deviation. **b**, Independent validation in four independent NSCLC cohorts. Bars represent point estimates and error bars indicate 95% bootstrap CIs (n = 1,000 resamples). P values were calculated using two-sided Wilcoxon signed-rank tests.

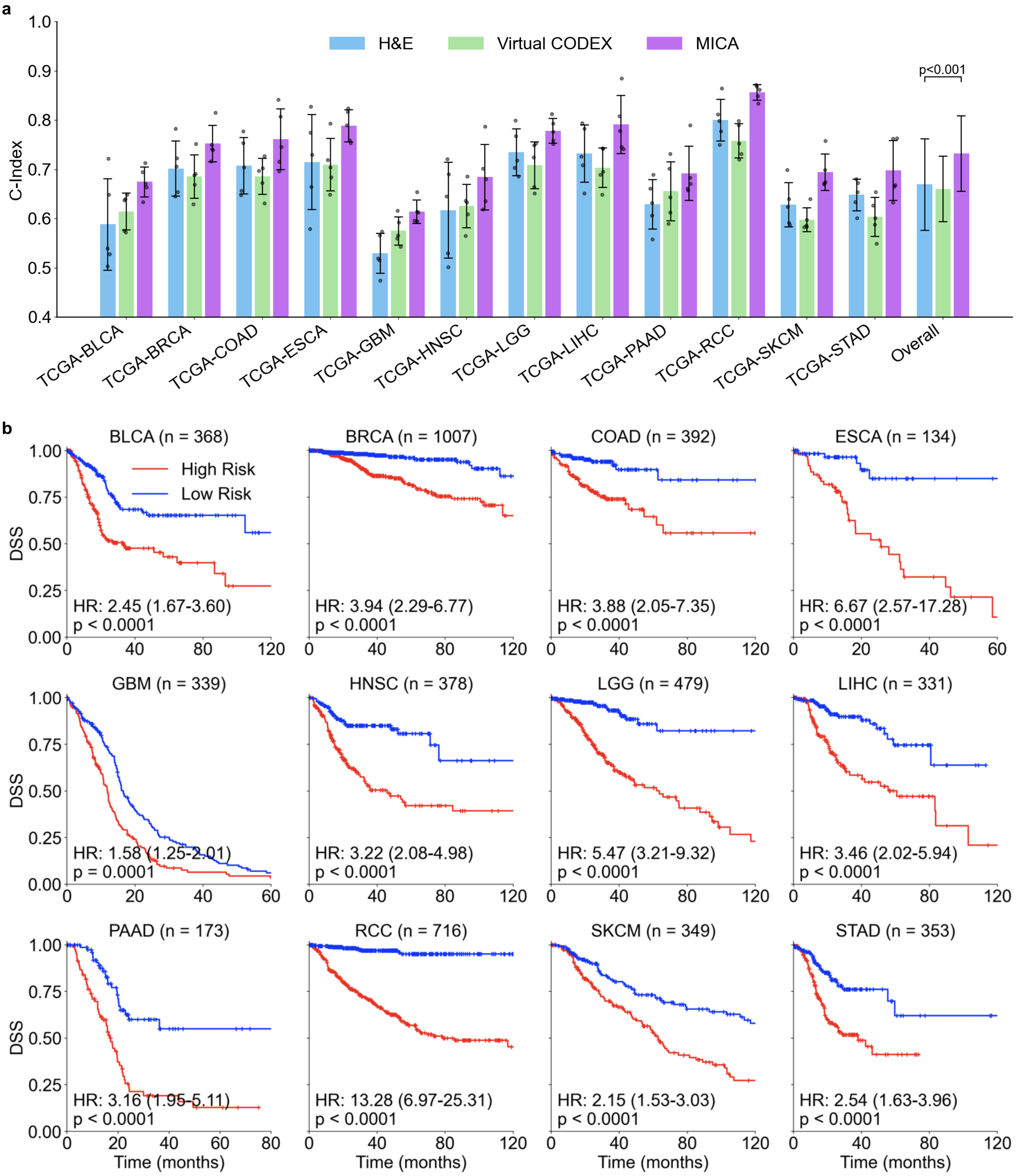

Supplementary Fig. 15 | MICA improves pan-cancer prognosis prediction. **a**, C-index comparison of H&E-only (MUSK), virtual CODEX-only, and MICA models. MICA outperforms both baselines across all cancer types. Bars represent the mean c-index across five-fold cross-validation for 12 TCGA cancer types; dots show individual folds and error bars indicate standard deviation. **b**, Kaplan–Meier curves for 12 TCGA cancer types using MICA-predicted risk groups. P values were calculated using two-sided Mann–Whitney U tests (**a**) and two-sided log-rank tests (**b**); no multiple-comparison adjustment.

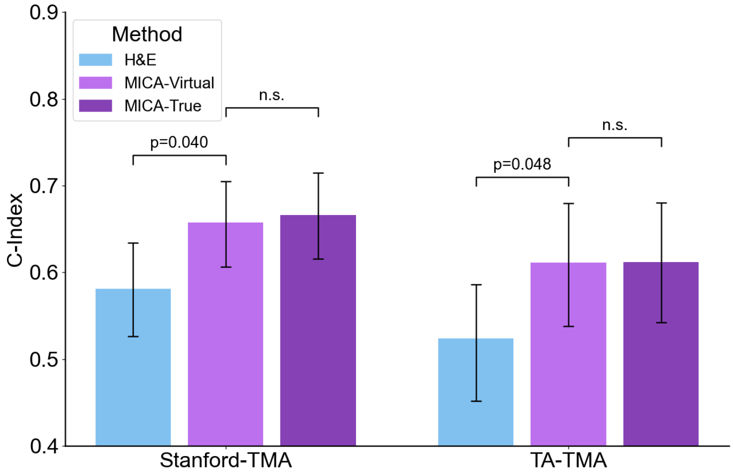

Supplementary Fig. 16 | HEX-derived virtual spatial proteomics matches true spatial proteomics for prognosis prediction. Comparison of c-index for H&E-only, MICA-Virtual (HEX), and MICA-True (true CODEX) models on Stanford-TMA (n = 226) and TA-TMA cohorts (n = 108). MICA-Virtual significantly outperforms H&E-only, and performs comparably to MICA-True. Bars represent point estimates and error bars indicate 95% bootstrap CIs (n = 1,000 resamples). P values were calculated by a two-sided paired bootstrap and were unadjusted. n.s., not significant.

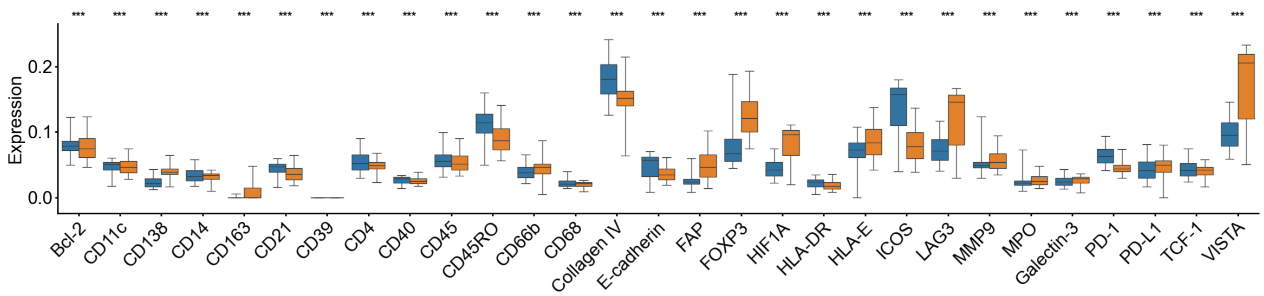

Supplementary Fig. 17 | Box plots comparing marker expression distributions between the high- and low-risk groups for remaining protein biomarkers (high-risk n = 26,519 patches; low-risk n = 26,416 patches). Center lines indicate medians; boxes span the interquartile range (25th–75th percentiles) and whiskers extend to the 5th–95th percentiles. The two-sided Mann–Whitney U test was used to assess the statistical significance between groups. ***, $P < 0.001$.

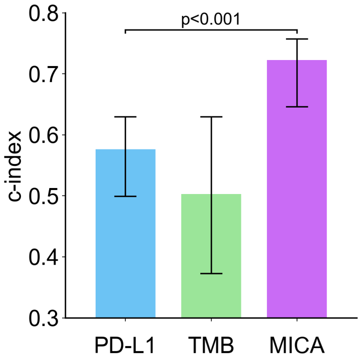

Supplementary Fig. 18 | Comparison of model performance for predicting PFS. Bar plots compare c-indices of models based on PD-L1, TMB, and MICA. Bars represent point estimates with 95% bootstrap CIs (n = 1,000 resamples) derived from aggregated risk predictions. Statistical significance was evaluated using a two-sided Mann–Whitney U test.

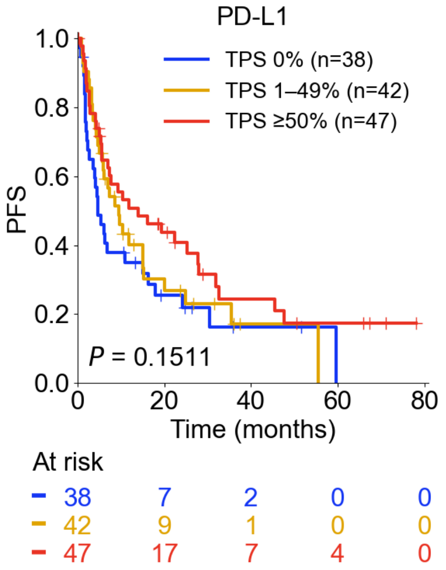

Supplementary Fig. 19 | Kaplan–Meier analysis stratified by PD-L1 expression. Patients were grouped by PD-L1 expression levels, and survival differences were assessed using a two-sided log-rank test.

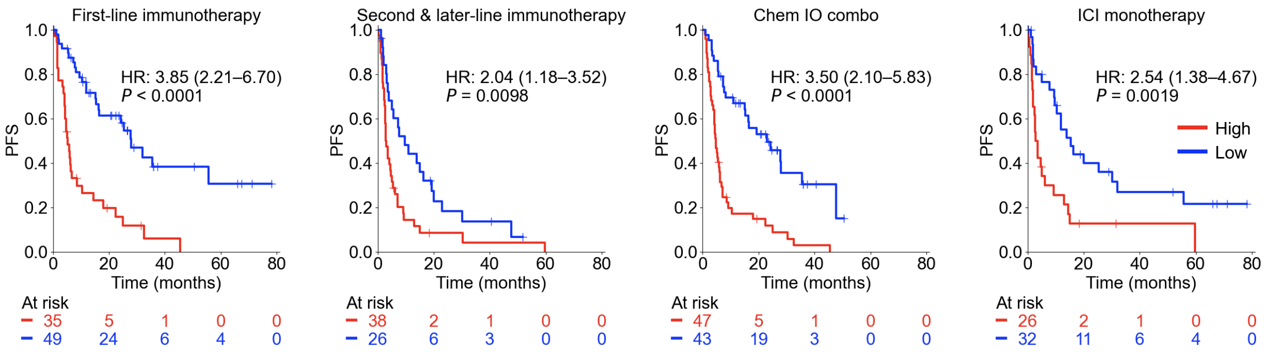

Supplementary Fig. 20 | Kaplan–Meier analysis of MICA risk groups in Stanford-IO subgroups. MICA significantly stratified patients by PFS across clinical subgroups. P values were calculated using two-sided log-rank tests.

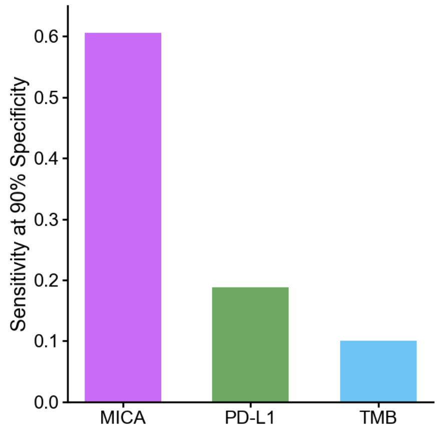

Supplementary Fig. 21 | Sensitivity at fixed specificity for predicting immunotherapy response. Comparison of MICA, PD-L1, and TMB for predicting objective response at 90% specificity.

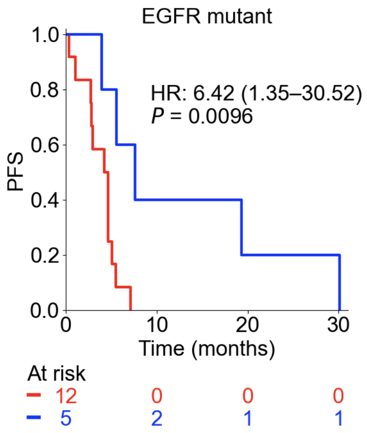

Supplementary Fig. 22 | Kaplan–Meier analysis of MICA risk groups in Stanford-IO subgroups defined by EGFR. MICA significantly stratified patients by PFS across clinical subgroups. P values were calculated using two-sided log-rank tests.

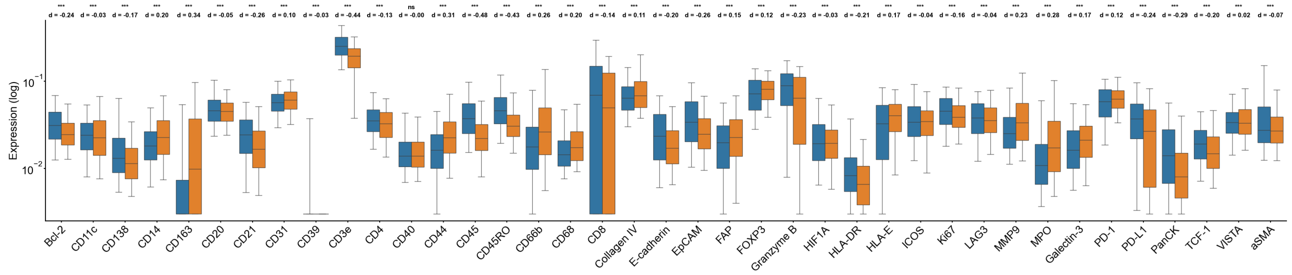

Supplementary Fig. 23 | Box plots comparing marker expression distributions between the high- and low-risk groups for all protein biomarkers (high-risk n = 2,967 patches; low-risk n = 2,986 patches). Center lines indicate medians; boxes span the interquartile range (25th–75th percentiles) and whiskers extend to the 5th–95th percentiles. Statistical significance was assessed using the two-sided Mann–Whitney U test. Effect sizes were calculated using rank-biserial correlation, providing a standardized measure of group differences. ***, $P < 0.001$, ns, not significant.

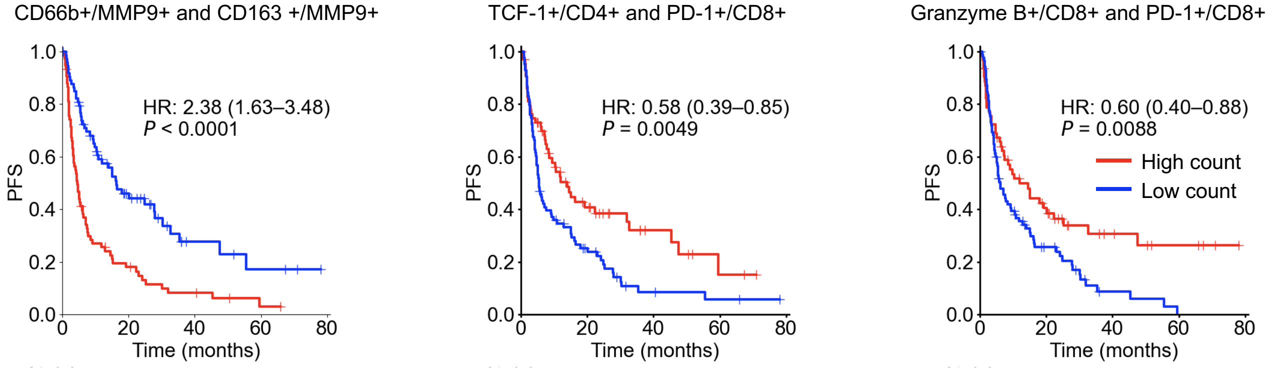

Supplementary Fig. 24 | Kaplan–Meier analysis stratified by SCS counts in Stanford-IO subgroups. Patients were stratified into high- and low-count groups based on the number of tiles positive for each SCS; *P* values were calculated by two-sided log-rank test.

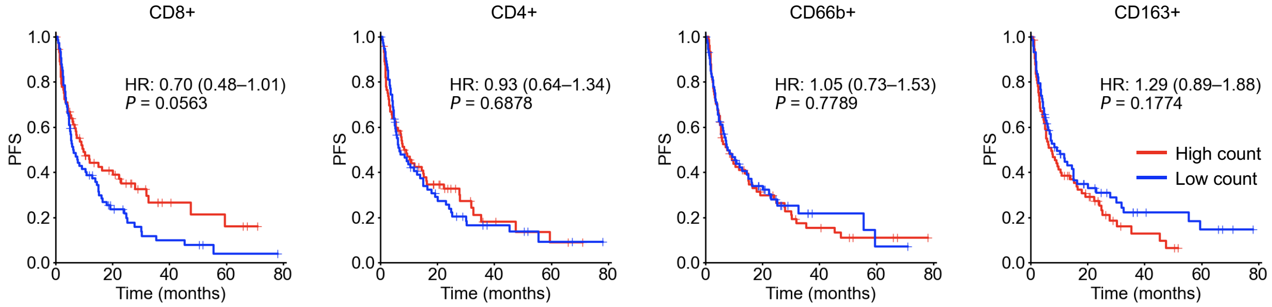

Supplementary Fig. 25 | Kaplan–Meier analysis stratified by single-biomarker expression in Stanford-IO subgroups. Patients were stratified into high- and low-count groups based on the number of tiles positive for each individual biomarker; *P* values were calculated by two-sided log-rank test.

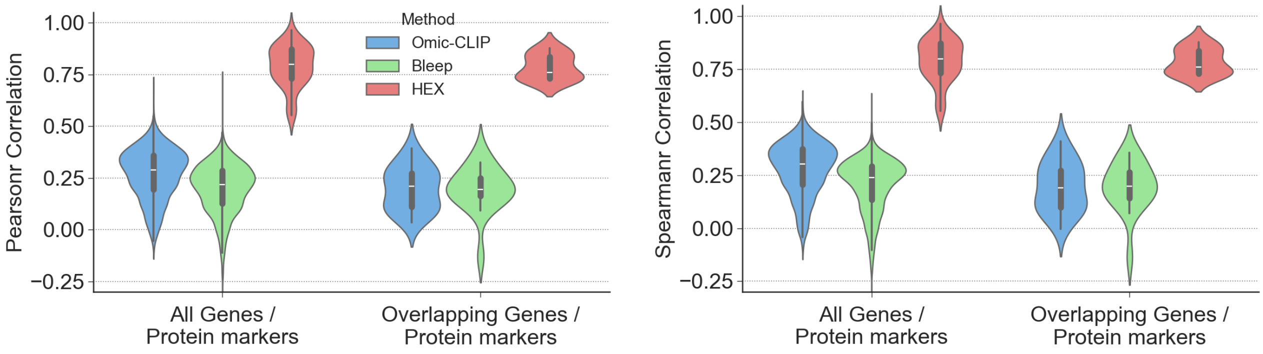

Supplementary Fig. 26 | Comparison of prediction accuracy between HEX and spatial transcriptomics (ST)-based models. Violin plots show Pearson (left) and Spearman (right) correlations for HEX, BLEEP, and Omic-CLIP. “All genes/protein markers”: 2,000 genes (ST) vs 40 protein markers (HEX); “Overlapping genes/protein markers”: 18 matched genes (ST) vs 11 markers (HEX). Violins depict the density of correlation values; embedded boxes show the median (center line) and interquartile range (25th–75th percentiles), with whiskers extending to 1.5x interquartile range.

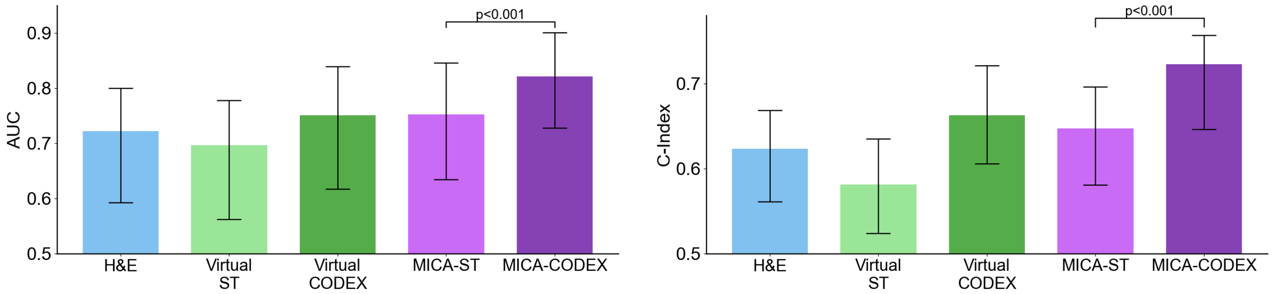

Supplementary Fig. 27 | Comparison of clinical utility between virtual spatial transcriptomics and virtual proteomics. Performance of MICA models trained on H&E combined with virtual ST or virtual CODEX in the Stanford-IO cohort (n = 148 patients). Left: immunotherapy response prediction (AUC). Right: progression-free survival prediction (c-index). MICA-CODEX significantly outperformed MICA-ST in both tasks. Bars represent point estimates (AUCs in left; c-indices in right) and error bars indicate 95% bootstrap CIs (n = 1,000 resamples) derived from aggregated risk predictions. Statistical significance was assessed using two-sided Wilcoxon signed-rank tests.

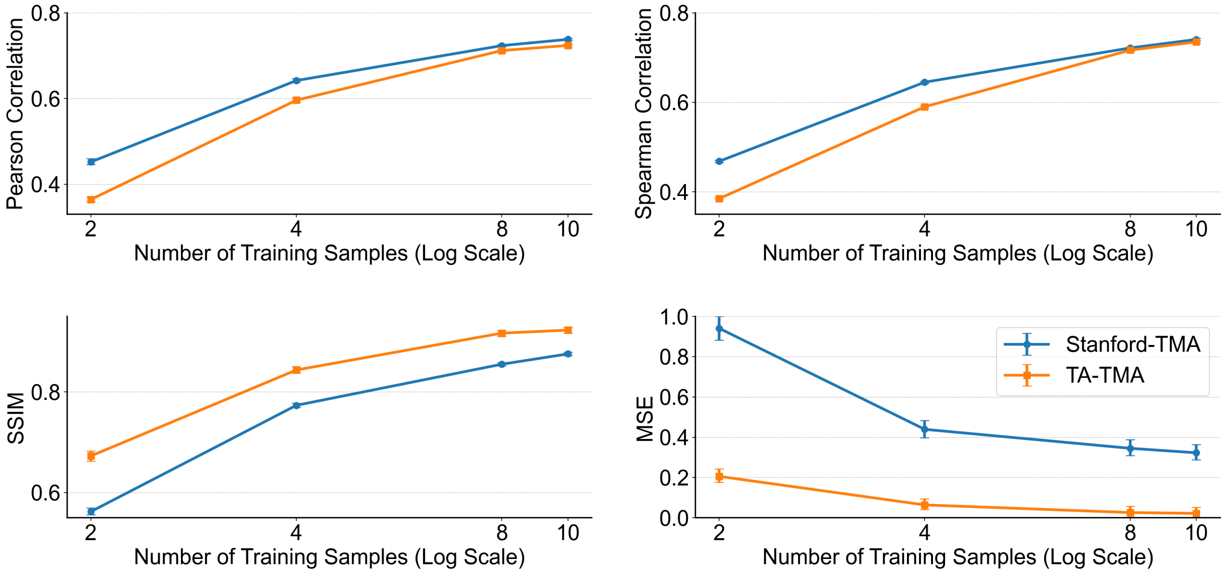

Supplementary Fig. 28 | Effect of training dataset size on HEX performance. HEX was trained on 2, 4, 8, and 10 WSIs and evaluated on the Stanford-TMA (n = 226) and TA-TMA (n = 108) cohorts. Performance was assessed using Pearson correlation, Spearman correlation, SSIM, and MSE. Points denote point estimate and error bars indicate 95% bootstrap CIs (n = 1,000 resamples).

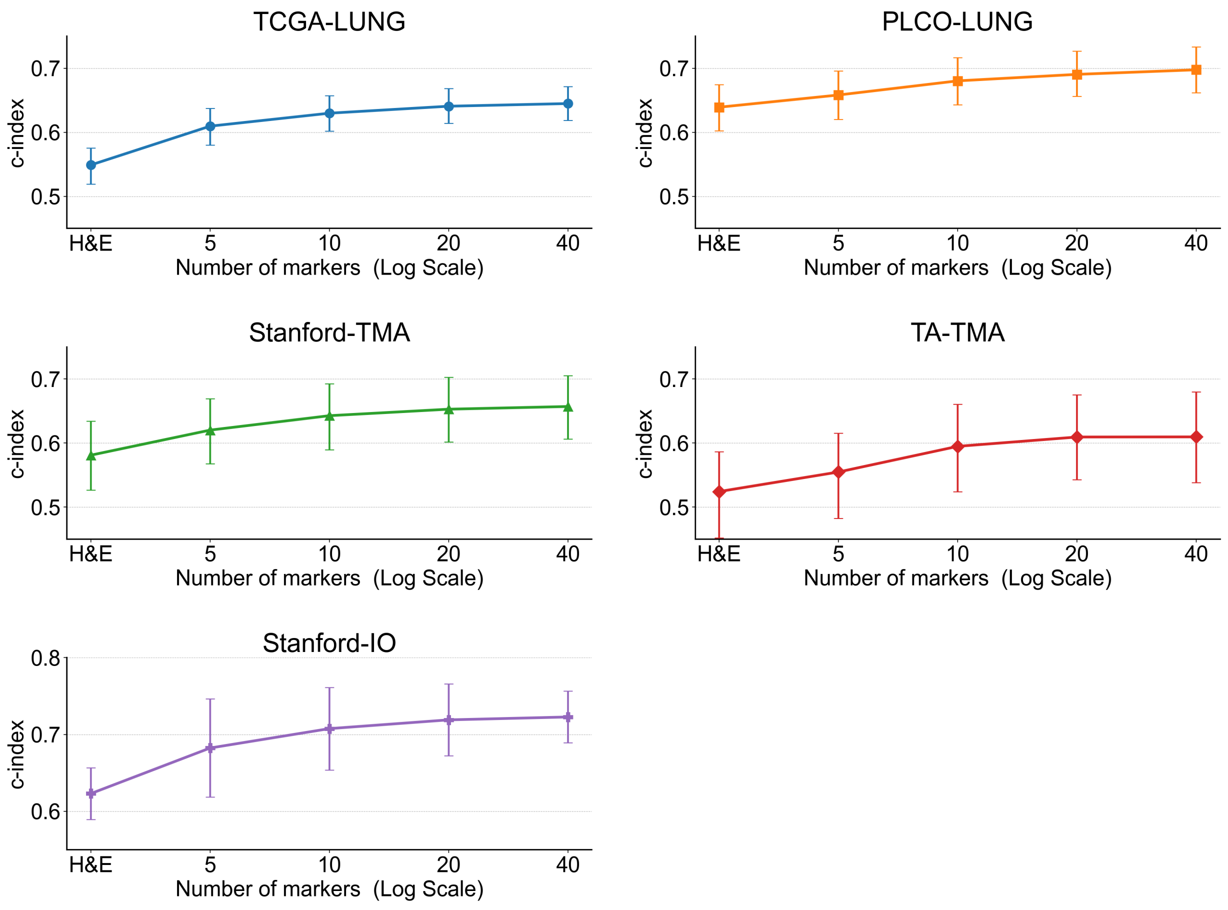

Supplementary Fig. 29 | Effect of marker panel size on prognostic performance. MICA was trained with 5, 10, 20, and 40 HEX-inferred markers and evaluated across five cohorts. Prognostic accuracy (c-index) improved with panel size but showed diminishing returns beyond 20 markers. Points denote point estimates and error bars indicate 95% bootstrap CIs (n = 1,000 resamples).

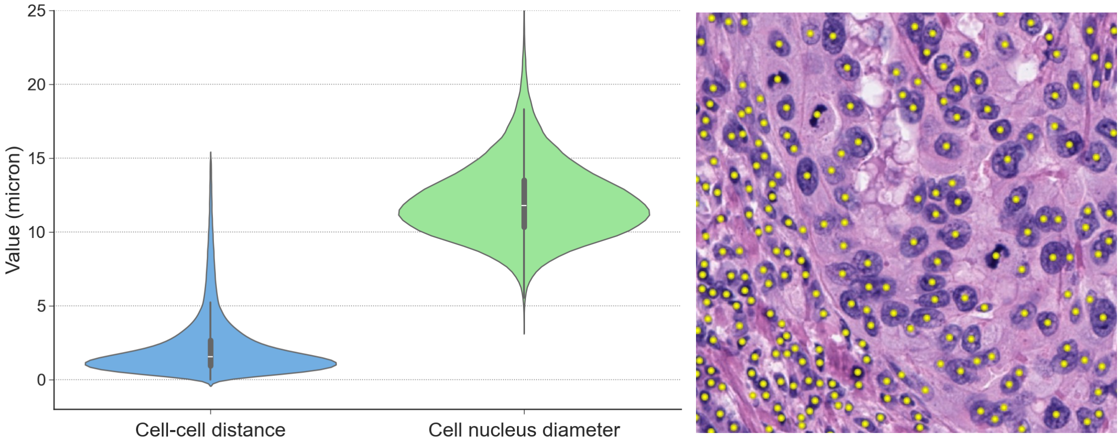

Supplementary Fig. 30 | Validation of H&E–CODEX registration accuracy. Left: Violin plots showing the distribution of minimum distances between H&E and mapped CODEX cell centroids compared to CODEX cell diameters (n = 13,084,594 cells). Embedded boxes show the median (center line) and interquartile range (25th–75th percentiles), with whiskers extending to 1.5x interquartile range. Right: Representative H&E image with mapped CODEX nuclei centroids overlaid in yellow.

5. Supplementary Tables

Supplementary Table 1: Refer to separate spreadsheet files

Supplementary Table 2 | Clinical characteristics of the Stanford-TMA cohort.

Stanford-TMA	
**Sex**	
Female	120 (53%)
Male	105 (47%)
Unknow	1 (0%)
**Age (median [IQR])**	67 (59-75)
**Histology**	
Adenocarcinoma	168 (74%)
Squamous cell carcinoma	58 (26%)
**T-stage**	
T1	61 (27%)
T2	113 (50%)
T3	31 (14%)
T4	12 (5%)
Unknow	9 (4%)
**Lymph node metastasis**	
N0	137 (61%)
N1	42 (19%)
N2	29 (13%)
Nx / missing	18 (8%)
**Cancer Stage**	
I	107 (47%)
II	80 (35%)
III	37(16%)
IV	1 (0%)
Unknown	1 (0%)
**Grade**	
I	37 (16%)
II	113 (50%)
III	64 (28%)
IV	1 (0%)
Unkonwn	11 (5%)

Supplementary Table 3 | Clinical characteristics of the TA-TMA cohort.

	TA-TMA
**Sex**	
Female	34 (32%)
Male	74 (69%)
**Age (median [IQR])**	56 (51-62)
**Histology**	
Adenocarcinoma	55 (51%)
Squamous cell carcinoma	53 (49%)
**T-stage**	
T1	3 (3%)
T2	88 (81%)
T3	17 (16%)
**Lymph node metastasis**	
N0	63 (58%)
N1	40 (37%)
N2	5 (5%)
**Metastasis**	
M0	108 (100%)
**Cancer Stage**	
I	49 (45%)
II	45 (42%)
III	14 (13%)
**Grade**	
I	3 (3%)
II	46 (43%)
III	58 (54%)
Unkonwn	1 (0%)

Supplementary Table 4 | Clinical characteristics of the Stanford-IO cohort.

	Stanford-IO
**Sex**	
Female	62 (42%)
Male	86 (58%)
**Age (median [IQR])**	70 (63-76)
**Smoking**	
Yes	100 (68%)
No	47 (32%)
Unknown	1 (1%)
**Histology**	
Adenocarcinoma	122 (82%)
Squamous cell carcinoma	20 (14%)
Others	6 (4%)
**PD-L1 TPS**	
0%	38 (26%)
1-49%	42 (28%)
≥ 50%	47 (32%)
Unknown	21 (14%)
**Line of therapy**	
1L	84 (57%)
2L	48 (32%)
3L+	16 (11%)
**Concurrent chemotherapy**	
Yes	90 (61%)
No	58 (39%)
**CNS Metastases**	
Yes	58 (39%)
No	78 (53%)
Unknown	12 (8%)
