# Supplementary material for: AI-enabled virtual spatial proteomics from histopathology for interpretable biomarker discovery in lung cancer
Source: Nat Med. 2026 Jan 5;32(1):231–44. doi: 10.1038/s41591-025-04060-4 (PMC12823406; doi:10.1038/s41591-025-04060-4)
Supplement: Supplementary file 2 — Reporting Summary [file 41591_2025_4060_MOESM2_ESM.pdf]

nature portfolio

Corresponding author(s): Ruijiang Li

Last updated by author(s): Sep 22, 2025

Reporting Summary

Nature Portfolio wishes to improve the reproducibility of the work that we publish. This form provides structure for consistency and transparency in reporting. For further information on Nature Portfolio policies, see our Editorial Policies and the Editorial Policy Checklist.

Statistics

For all statistical analyses, confirm that the following items are present in the figure legend, table legend, main text, or Methods section.

n/a	Confirmed	
☐	☒	The exact sample size (*n*) for each experimental group/condition, given as a discrete number and unit of measurement
☐	☒	A statement on whether measurements were taken from distinct samples or whether the same sample was measured repeatedly
☐	☒	The statistical test(s) used AND whether they are one- or two-sided<br>*Only common tests should be described solely by name; describe more complex techniques in the Methods section.*
☐	☒	A description of all covariates tested
☐	☒	A description of any assumptions or corrections, such as tests of normality and adjustment for multiple comparisons
☐	☒	A full description of the statistical parameters including central tendency (e.g. means) or other basic estimates (e.g. regression coefficient) AND variation (e.g. standard deviation) or associated estimates of uncertainty (e.g. confidence intervals)
☐	☒	For null hypothesis testing, the test statistic (e.g. F, t, r) with confidence intervals, effect sizes, degrees of freedom and P value noted<br>*Give P values as exact values whenever suitable.*
☒	☐	For Bayesian analysis, information on the choice of priors and Markov chain Monte Carlo settings
☐	☒	For hierarchical and complex designs, identification of the appropriate level for tests and full reporting of outcomes
☒	☐	Estimates of effect sizes (e.g. Cohen's *d*, Pearson's *r*), indicating how they were calculated

Our web collection on statistics for biologists contains articles on many of the points above.

Software and code

Policy information about availability of computer code

Data collection	High-plex image acquisition was performed on the PhenoCycler 2.0 platform per manufacturer protocol. H&E images were obtained post-CODEX on a Leica Aperio AT2 scanner. All deep learning experiments were performed using Python (3.10.15).
Data analysis	Data analysis was performed using Python (3.10.15) and PyTorch (2.4.0+cu118), with key libraries including lifelines, scikit-learn, and matplotlib. Analyses utilized open-source toolkits such as MUSK, PALOM, DINOv2, CLAM, MCAT, and imbalanced-regression. Deep learning experiments were run on NVIDIA GPUs (L40S x8) with CUDA 11.8 and cuDNN 9.1 (Ubuntu 22.04). An open-source version of the code is available at https://github.com/lilab-stanford/HEX.

For manuscripts utilizing custom algorithms or software that are central to the research but not yet described in published literature, software must be made available to editors and reviewers. We strongly encourage code deposition in a community repository (e.g. GitHub). See the Nature Portfolio guidelines for submitting code & software for further information.

Data

Policy information about availability of data

All manuscripts must include a data availability statement. This statement should provide the following information, where applicable:

- Accession codes, unique identifiers, or web links for publicly available datasets
- A description of any restrictions on data availability
- For clinical datasets or third party data, please ensure that the statement adheres to our policy

The TCGA clinical and histopathology data analyzed in this study are publicly accessible via the NCI Genomic Data Commons (https://portal.gdc.cancer.gov) and cBioPortal (https://www.cbioportal.org). NLST whole-slide image (WSI) data are publicly available from The Cancer Imaging Archive (https://wiki.cancerimagingarchive.net). PLCO data can be requested through the Cancer Data Access System (https://cdas.cancer.gov/learn/plco). Data from the Stanford-WSI, Stanford-TMA, TA-TMA, and Stanford-IO cohorts are subject to institutional restrictions due to patient confidentiality. Researchers may request access to these datasets from the corresponding author by submitting a brief research proposal and completing a data use agreement, with usage restricted to academic, non-commercial research purposes.

Research involving human participants, their data, or biological material

Policy information about studies with human participants or human data. See also policy information about sex, gender (identity/presentation), and sexual orientation and race, ethnicity and racism.

Reporting on sex and gender	Not applicable.
Reporting on race, ethnicity, or other socially relevant groupings	Socially relevant characteristics of the study participants (when available) are provided in Supplementary Table 2, 3, and 4.
Population characteristics	Detailed population characteristics for the in-house datasets are provided in Supplementary Table 2, 3, and 4.
Recruitment	No patient recruitment was necessary for the use of whole-slide images retrospectively.
Ethics oversight	This retrospective study was approved by the institutional review board at Stanford University.

Note that full information on the approval of the study protocol must also be provided in the manuscript.

Field-specific reporting

Please select the one below that is the best fit for your research. If you are not sure, read the appropriate sections before making your selection.

☒ Life sciences ☐ Behavioural & social sciences ☐ Ecological, evolutionary & environmental sciences

For a reference copy of the document with all sections, see nature.com/documents/nr-reporting-summary-flat.pdf

Life sciences study design

All studies must disclose on these points even when the disclosure is negative.

Sample size	A total of 2,298 patients with histologically confirmed non-small cell lung cancer (NSCLC) were included across seven independent cohorts. For prognosis prediction, five cohorts comprising 2,150 patients with available H&E-stained whole-slide images (WSIs) and clinical outcome data were used: the National Lung Screening Trial (NLST), The Cancer Genome Atlas (TCGA), the Prostate, Lung, Colorectal, and Ovarian Cancer Screening Trial (PLCO), and two tissue microarray cohorts—Stanford-TMA and TA-TMA—used for external validation. For immunotherapy response analysis, the Stanford-IO cohort included 148 patients with advanced NSCLC treated with PD-1 or PD-L1 immune checkpoint inhibitors, with corresponding H&E slides from pretreatment biopsies. Cohort-specific clinical characteristics and outcome definitions are provided in Supplementary Tables 2–4.
Data exclusions	Samples were excluded based on predefined clinical and technical criteria. Specifically, cases were removed if they were not histologically confirmed as NSCLC, had poor-quality H&E slides unsuitable for analysis, or lacked recurrence-free survival or progression-free survival data. One additional case was excluded from the immunotherapy cohort due to not meeting the advanced NSCLC criteria. All exclusions were made prior to model training or evaluation.
Replication	Model performance was evaluated using both cross-validation and independent validation. For cross-validation, models were trained and tested using five-fold splits, and all experiments were repeated five times to assess reproducibility. For independent validation, models trained on one cohort were evaluated on external datasets without retraining. All replication attempts were successful and showed consistent results across runs and datasets.
Randomization	For five-fold cross-validation, patients were randomly assigned to training and validation sets in each fold.
Blinding	Blinding was not necessary because the experiments were based on digitized histology slides.

Reporting for specific materials, systems and methods

We require information from authors about some types of materials, experimental systems and methods used in many studies. Here, indicate whether each material, system or method listed is relevant to your study. If you are not sure if a list item applies to your research, read the appropriate section before selecting a response.

Materials & experimental systems

n/a	Involved in the study	
☐	☒	Antibodies
☒	☐	Eukaryotic cell lines
☒	☐	Palaeontology and archaeology
☒	☐	Animals and other organisms
☒	☐	Clinical data
☒	☐	Dual use research of concern
☒	☐	Plants

Methods

n/a	Involved in the study	
☒	☐	ChIP-seq
☒	☐	Flow cytometry
☒	☐	MRI-based neuroimaging

Antibodies

Antibodies used	Detailed in Methods.
Validation	All antibodies used are commercially available and have been validated by the corresponding vendors

Plants

Seed stocks	*Report on the source of all seed stocks or other plant material used. If applicable, state the seed stock centre and catalogue number. If plant specimens were collected from the field, describe the collection location, date and sampling procedures.*
Novel plant genotypes	*Describe the methods by which all novel plant genotypes were produced. This includes those generated by transgenic approaches, gene editing, chemical/radiation-based mutagenesis and hybridization. For transgenic lines, describe the transformation method, the number of independent lines analyzed and the generation upon which experiments were performed. For gene-edited lines, describe the editor used, the endogenous sequence targeted for editing, the targeting guide RNA sequence (if applicable) and how the editor was applied.*
Authentication	*Describe any authentication procedures for each seed stock used or novel genotype generated. Describe any experiments used to assess the effect of a mutation and, where applicable, how potential secondary effects (e.g. second site T-DNA insertions, mosiacism, off-target gene editing) were examined.*
